# Supplementary material for: Are carbon emissions trading and green financial instruments synergistic? -Comprehensive quantitative research based on content analysis
Source: PLoS One. 2024 Mar 7;19(3):e0298601. doi: 10.1371/journal.pone.0298601 (PMC10919601; doi:10.1371/journal.pone.0298601)
Supplement: S1 Data — (ZIP) [file pone.0298601.s001.zip › Data/地级市Z_F工作报告.zip!/╦─┤¿/├╝╔╜╩╨/└┤╫╘╣1⁄2╓┌║┼í╛┬φ┐╦╩2╛▌═°í┐_2019.docx]

各位代表：

我代表市人民政府，向大会报告工作，请予审议，并请市政协委员提出意见。

一、2018年工作回顾

过去一年，是眉山追赶跨越极不平凡的一年。一年来，面对宏观环境复杂多变、区域竞争不断加剧，我们坚持以习近平新时代中国特色社会主义思想为指导，全面贯彻党的十九大精神，深入学习贯彻习近平总书记对四川工作系列重要指示精神，在省委、省政府和市委的坚强领导下，认真落实省委十一届三次、四次全会和市委四届十一次全会部署，紧扣市委治眉兴眉“135”总体布局和“落实年”工作主题，克难奋进、爬坡过坎，较好完成了市四届人大四次会议确定的目标任务。

这一年，省委明确眉山建设开放发展示范市的新定位，新时代的眉山迎来了新的历史机遇。

这一年，眉山天府新区落实习近平总书记指示精神，按照“天府公园城·眉山创新谷”的定位，调整理顺体制机制，站在了高起点发展新的起跑线上。

这一年，成德眉资同城化首次上升为全省战略，成眉双方高频互动，签订合作协议30个，成眉合作驶入了快车道。

这一年，全市财政收支取得新突破，地方一般公共预算收入迈上100亿元台阶，基金收入迈上300亿元台阶，财政总支出迈上500亿元台阶。

这一年，建市以来最大工业项目，总投资443亿元的信利高端显示面板项目首条模组生产线正式投产，眉山工业发展基础更加坚实。

这一年，眉山乡村振兴经验受到国务院办公厅通报表扬，东坡区现代农业产业园名列全国第一批国家现代农业产业园之首，中法农业科技园荣获法中两国政府“创新合作奖”。

这一年，瓦屋山景区时隔六年全新开放，三苏祠博物馆荣获联合国教科文组织“世界文化遗产保护荣誉证书”，彭山江口古战场遗址入选“全国十大考古新发现”，文旅盛典不断点亮眉州大地。

这一年，区县和市级部门多项工作走在了全国全省前列，荣获上百项表彰与奖励，为眉山争得荣誉！

（一）经济质效明显提升。

发展态势稳中向好。全市地区生产总值1256.02亿元，增长7.5%。地方一般公共预算收入103.2亿元，增长10%。全社会固定资产投资完成1031.6亿元，增长12.7%。社会消费品零售总额496.4亿元，增长12%。城乡居民人均可支配收入分别为33697元、16563元，分别增长8.2%、8.9%。居民消费价格指数上涨1.6%，常住人口城镇化率提高1.55个百分点、达46.32%。一年来，全市上下齐心协力、苦干实干，主要经济指标高于上年、好于预期。

现代农业稳中提质。第一产业增加值增长3.8%。全年新建高标准农田20.6万亩，粮食总产量达123.8万吨，粮食安全市长责任制4项考核全省第一。新认定省级农业产业化龙头企业14个、中国特色农副产品之乡2个。新培育农民合作社和家庭农场930个，“三品一标”农产品达到259个。特色农业发展壮大，“味在眉山”销售收入达839.5亿元，其中泡菜收入181.5亿元、增长9.5%。青神县建成全国竹产业交易中心，国际（眉山）竹产业交易博览会成功举办，林竹产值达238亿元。新发展晚熟柑橘6.4万亩、达65万亩，眉山正成为全国最大晚熟柑橘生产基地。全年水果产值120亿元，增长18.8%。“东坡泡菜”“丹棱桔橙”“青神竹编”跻身2018中国区域品牌价值评价百强榜。眉山成功创建全省农产品质量安全监管示范市。养殖业稳步发展，主要畜禽规模化养殖比重42.6%，建成种养循环示范基地12个，华西、正大规模化畜禽养殖项目加快推进。成功承办全国蜂业大会，蜂蜜产量全省第一。

工业转型稳中提速。规上工业总产值达1395.6亿元，规上企业达593户，规上工业增加值增长9%，完成工业投资308.9亿元、增长21.4%，工业入库税金达到31亿元。落后产能加快淘汰，单位工业增加值综合能耗下降7%、水耗下降6%。方大炭素、康佳中国第二总部基地等一批先进制造业项目落地开工，陶氏化学、能投鼎盛等一批重大项目竣工投产，战略性新兴产业产值增长15%，“3135”产业产值达到1148.3亿元，占全市工业比重达82%。45户骨干企业产值358.6亿元，增长30.8%。金象赛瑞等3户企业通过国家级绿色工厂认证。新增规上工业企业51户，新增10亿元以上企业2户。上市公司发展强劲，千禾味业产值和入库税金分别增长21.8%、56.6%。盘活闲置企业100户，清理整理出闲置工业用地7550亩。为工业企业办理风险补偿贷款和应急转贷资金9.8亿元。争取甘孜州留存电21亿千瓦时，降低企业税收、用能费用等成本36.5亿元。

三产发展稳中趋优。第三产业增加值增长9.5%。商贸物流快速发展，电子商务网络交易额增长38.7%，邮政业务总量增长62.6%，电信业务总量增长34.4%。外贸进出口总额达到6.6亿美元，增长140%。成功获批国家外贸转型升级基地、海峡两岸冷链物流示范市。中车眉山公司海外销售突破1亿美元。金融业快速发展，全省率先成立不动产“金融超市”，光大银行眉山分行完成筹建，全市存款余额2094.5亿元，增长6.6%，贷款余额1043.1亿元，增长20.6%，贷存比上升5.8个百分点。高端旅游迅速发展，乐高乐园、恒大童世界、七里坪太阳季等项目完成前期工作，新增国家级森林康养基地1个、省级3个，洪雅成为中国宜居养生之都、四川省乡村旅游强县，丹棱跻身全国十大“最美探春地”，全年旅游人数4790.7万人次、增长10%，实现旅游收入404.3亿元、增长13.4%。乡村旅游蓬勃发展，涌现出一批深受游客欢迎的特色旅游产品，全年接待游客1640万人次，实现收入101.1亿元。

（二）开放步伐明显加快。

招商引资再创新高。精心组织眉山企业参与招商小分队赴欧美、日韩、东南亚等12个国家海外招商、推销产品，到港澳台、珠三角、长三角等20余个地区专场推介，组织参加中国国际进口博览会、西博会、农博会、广交会、香港食品展等国家、省重大会展活动，引进中日国际康养城等“三类500强”或投资10亿元以上重大项目36个，总投资3883亿元，全年招商引资到位资金675亿元。外商投资到位资金1亿美元，新设外资企业10家。

成眉同城全面提速。“成眉124重点通道”加快推进，剑南大道黄龙溪段打通“最后一公里”，岷东大道永寿以北段全线通车。视高至成都兴隆湖全省首条跨市域公交班车正式开通。成眉高铁由29列/天加密至51列/天。成眉市域铁路S5、S14线前期工作积极推进。投资50亿元的成都美富特绿色表面处理中心项目落户青神，全年引进成都工业企业签约项目42个，其中10亿元以上项目8个。西南联合产权交易所眉山分所成功挂牌。与成都共建共享乡村振兴“七大共享平台”。住房公积金实现缴存同基数、转接无障碍。医疗、教育等合作加快推进，华西第二医院眉山妇女儿童医院开工建设，省人民医院入驻黑龙滩。

开放影响显著扩大。东坡区眉山会展中心建成投用。成功举办第十六届中国食品安全年会暨第十届中国泡菜食品国际博览会、北京大学全球校友会等大型节会活动上百场，近两千名国外政要汇聚眉山，活动次数、规格、影响均破眉山纪录。西成客专“苏东坡”号驰骋秦巴大地，东坡国际半程马拉松赛央视直播，“东坡泡菜”广告亮相纽约时代广场。与70多个国家签订经贸合作协议，与8个国外城市建立友好关系。中外媒体频频“点赞”眉山，开启了从“眉山看世界”到“世界看眉山”的新篇章！

（三）改革创新明显进展。

改革力度持续加大。全面完成10项中省改革试点、106项市级改革任务。投融资改革深入推进，实现直接融资101.8亿元。财税改革稳步推进，全年税收收入63.2亿元，税占比达61.2%，提升6.1个百分点。向上争取资金200亿元以上，成功争取发行地方政府新增专项债券31.6亿元、全省第六，有效用于民生改善、旧城改造、园区升级、乡村振兴等重点资金需求。积极加强政府债务管理，债务余额严格控制在省核定限额内并持续减少。全国殡葬综合改革试点深入推进。事业单位登记管理经验全国推广。农村土地“四步流转”等4条改革经验入选全省全面深化改革典型案例。重组眉山发展公司、市资产经营公司、眉山天府新区投资集团有限公司三大国有企业集团，全市国有资产总额达到1322.3亿元，增长14.7%。全面推进全程电子化工商登记和简易注销登记改革，新增个体工商户和私营企业分别为27025户、4664户，分别增长17.1%、8.9%。引导支持60户民营企业建立现代企业制度，民营经济增加值增长7.6%。

创新动能持续提升。新增国家高新技术企业5户，销售上2亿元高新技术企业10户，全市高新技术产业主营业务收入达324亿元。眉山经开区成功获批省级高新技术开发区。甘眉园区等5个园区新晋为省级经济开发区。国家新药药效评价中心建成投运、“西部药谷”成为全国药品上市许可持有人试点园区。新培育省级企业技术中心8户、省级技术创新示范企业2户。新建成省级工程技术研究中心、科技企业孵化器、众创空间6个。实施重大科技成果转化示范项目30项，4项科技成果获省科技进步奖，申请发明专利770件，有效发明专利累计达698件。青少年科技创新大赛获全国二等奖2项，省级一等奖10项。新培育国家地理标志保护产品2个、四川名牌企业50户，7户企业通过国际食品安全保证体系认证。开展“四川省高级专家（博士后）走进眉山”智力服务活动，创新柔性引才模式。与省“千人计划”专家联谊会合作共建四川眉山新材料产业示范园。全市规上企业研发投入达5亿元，增长15%。

（四）城乡面貌明显变化。

交通更通达。千亿交通大会战全年完成投资148亿元，再创历史新高。天府仁寿大道三绕至城区段、工业大道东坡区界至谢家段、太和大道简蒲眉山互通段、岷东新区至黑龙滩景区等4条快速通道建成通车。成乐高速扩容改造和环天府新区快速通道等5条道路加快建设。成宜高速和红星路南延线等3条快速路开工建设。岷江航电、洪雅直升机基地进展顺利。公交都市创建稳步推进，中心城区新增新能源公交车40辆，眉山天府新区实现全域公交化。

城市更宜居。完成城镇基础设施投资221亿元，新增建成区8.5平方公里。棚户区改造4404套、搬迁入户3520套。东坡岛拆迁居民安置顺利完成。出台既有住宅增设电梯政策，全年新装电梯15部。新（改）建城区公厕291座，中心城区新增停车位7622个。全国文明城市、国家卫生城市、国家森林城市创建深入开展，取得阶段性成果。

乡村更美丽。“清洁乡村·美丽眉山”行动全面展开，农村生活垃圾分类治理有力推进，无害化处理率94%，完成农村公共厕所改造166座、“四好”农村路466公里，新（改）建农村公路368公里，改造农村土坯房8447户。新增省级试点镇13个，新建成幸福美丽新村137个，34个村被评为全国第一批“绿色村庄”。丹棱县被列为全国农村人居环境整治试点县，幸福古村入选全国100个特色村庄。

（五）生态环境明显改善。

天越来越蓝。PM2.5平均浓度下降19.6%；环境空气质量优良率达79.3%，提升5个百分点，优良天数达289天，改善幅度均居全省第一。

地越来越净。农业面源污染防治加强，化肥、农药使用量分别下降11%、3.5%，秸秆综合利用率达92.7%，规模化畜禽养殖粪污利用率82.2%。建成生活垃圾无害化处理设施3个，新增垃圾中转站64个。工业固体废物及危险废物综合处置利用率100%，土壤环境质量保持稳定。

山越来越绿。完成营造林18.3万亩，建成城区公园绿地2891亩，新增乡村公共绿地2100亩，城乡绿化覆盖率达55.2%，森林覆盖率达49.5%，成功创建省级森林城市，建成全国生态文化村1个、全国最美森林小镇2个、省级森林小镇3个、森林村庄26个。瓦屋山省级自然保护区和周公河珍稀鱼类省级自然保护区内46座小水电站、52个矿硐全面关停，矿区植被恢复率100%，受到中央生态环保督察组高度赞扬。

水越来越清。加快实施全域污水处理工程，全面启动建设县、乡污水处理设施205座，完成配套管网400公里，第二污水处理厂完成主体工程。持续开展“清河、护岸、净水、保水”行动，整治河流、沟渠1800公里，纳入国、省考核的8个断面连续五个月消除劣V类。通惠河综合治理工程全面启动，截污主管全面贯通，河道清淤全面完成，长期困扰中心城区群众的“臭水沟”得到彻底整治！新增和恢复水域面积26.1平方公里，饮用水源地保护区内的排污口全部取缔，“引青入丹”工程即将通水，县级及以上饮用水源地水质达标率100%。完成整改中央、省环保督察“回头看”反馈问题71件，解决了一批长期想解决但没有解决好的问题。洪雅县获国家生态文明建设示范县称号，仁寿县获全国绿色发展百强县称号。一幅“天蓝地绿、山清水秀”的画卷正在眉山大地绘就！

（六）民生福祉明显增进。

民生投入不断增加。坚持新增财力绝大部分用于改善民生，完成民生投入488亿元，占总支出的85.5%。全市十大民生工程129个项目和20件民生大事全面完成，投入资金171.1亿元，增长11.6%。

教体发展迎来突破。教育基础设施显著改善，新（改）建校舍7.8万平方米，22所公办幼儿园开工建设，14所城区学校升级扩容，北外附属东坡外国语学校和尤文图斯足球学院培训中心开工建设，全面实现县域内义务教育基本均衡。教育质量稳步提升，高考本科上线8170人，东坡小学、青神学道街小学德育工作经验被评为全国典型，丹棱中学生李蝶获全国最美中学生称号。在眉高校总数达12所。体育事业加快发展，全民健身活动中心、青少年体育活动中心建成投用。竞技体育水平快速提升，国家乡村公园山地自行车挑战赛、全国皮划艇青少年锦标赛等重大比赛在眉举办，第十三届省运会获3金11银，第九届省残运会获22金15银。眉山启明星龙舟队参加瑞士国际龙舟赛勇夺两项冠军。

文化事业亮点纷呈。成功举办东坡文化国际学术高峰论坛暨四川音乐周，俄罗斯、奥地利交响乐团访问眉山。话剧《苏东坡》登上国家大剧院，江口沉银考古成果走进国家博物馆。市美术馆新馆开馆。文化惠民日益深入，免费放映农村公益电影1.3万余场次，建成全省首批“高清四川智慧广电”示范市。丹棱县引导民间众筹文化院坝建设获国家第三批公共文化服务体系示范项目。4个新项目列入省级非遗名录，青神竹编大师陈云华入选第五批国家级非遗项目代表性传承人。省第九届中小学生艺术节获一等奖24个，创历史新高。

健康眉山加快推进。市儿童福利院、残疾儿童康复中心投入使用。市人民医院二期工程、市中医医院和彭山区、洪雅县、青神县中医医院主体完工。眉山药科职业学院建成招生。深化医疗卫生体制改革，建成医联体23个，推进分级诊疗，实现县域内就诊率90%。推进公立医院改革，全面实现药品、医用器材全部零差率销售。加强传染病、地方病防控，探索建立慢性病管理“眉山模式”。落实计生惠民政策，惠及38.9万人。12家医院接入国家平台，13家医院开通省内异地普通门诊。城乡居民电子健康档案建档率97.5%，每年免费为65岁以上老年人健康体检一次。母婴安全保障工作位居全省前列，创建全市首个省级儿童早期发展示范基地。免费为8.7万名农村妇女进行“两癌”筛查。建成医养结合机构9家，床位2555张。落实抗癌药降价政策，地中海贫血、血友病纳入医保特殊疾病门诊管理。

养老事业升级提档。完成农村敬老院“公建民营”7所，建成服务标准化示范敬老院6所，改造农村养老服务中心48所，为8.4万名困难失能老人提供居家养老服务，农村养老“眉山模式”全省推广。

脱贫攻坚决战决胜。全年减少贫困人口11000人，15852人易地搬迁入住，全市10.94万建档立卡贫困人口全部脱贫，被省委、省政府评为脱贫攻坚先进市。加强对口帮扶，茂县脱贫摘帽，金川县达到摘帽标准，眉山市荣获省对口帮扶藏区彝区工作先进集体。

就业社保不断加强。新增城镇就业3.5万人，城镇登记失业率3.94%。引导大学生创新创业2102人，返乡创业10506人，发放创业担保贷款2563万元。1000名“苏小妹”通过培训被选聘京沪。仁寿县双创服务中心成为省级大学生创新创业园区。社会保险不断扩面提质。城乡低保标准分别提高8.3%、15.8%。城乡医疗救助困难群众23.6万人次。为7.8万建档立卡特困人员代缴养老保险。对1.5万名农村留守儿童和城乡困境儿童开展关爱保护，11.4万残疾人受到针对性的个性服务。

社会治理创新推进。深化平安眉山建设，扎实推进扫黑除恶专项斗争，全市刑事、治安案件立案数双双下降，成功侦办东坡“3·22”黑社会性质组织犯罪、“鑫圆系”特大网络传销等大要案件，被中外城市竞争力研究院评为中国最安全城市30强。民警王涛、廖弦用生命捍卫群众安全，被公安部、省委追授“一级英模”“四川省优秀共产党员”。安全生产稳步推进，生产安全事故起数、死亡人数分别下降19%、17.4%。建成全省首个市、县、乡三级救灾资金和物资管理信息系统，成功应对历史罕见的特大暴雨洪涝灾害，地质灾害连续十年“零死亡”。食药监管不断加强，查处食品药品违法案件1673件。深化社会稳定风险评估机制，妥善化解信访积案难案222件，信访数量下降8.9%。创新研发“矛盾纠纷多元化解一体化平台”，推动出台四川首部行政执法与刑事司法衔接决定，经验得到最高人民法院和最高人民检察院推广。加快公共法律服务体系建设，劳动人事争议案件调解成功率、结案率全省第一。在全国建立12个农民工服务站，为农民工追回薪资339.5万元。创新推行便民自治摊点管理模式，网格管理工作质效全省第一。退伍军人罗永田被评为“中华慈善楷模”，入选中国好人榜。

各项事业全面发展。国家安全、国防动员、双拥、人民防空、消防、保密、气象等工作扎实推进，民族、宗教、文史、档案、地方志、红十字会等工作不断加强。

（七）行政效能明显提高。

政务服务进一步优化。深入推进“互联网+政务服务”，市本级审批服务事项网上可办比例达89%，“最多跑一次”比例达91.7%，45项事项实现“零跑路”。创新实施“容缺审批重大事项”“红色代办”“模拟审批”，税务服务“全城通办”，审批时限提速30%以上。企业开办时间压缩至5个工作日内，项目从立项到施工许可压减至55个工作日内。

工作作风进一步严实。坚持勤俭办公，公务接待费、公车购置和运行维护费下降4.6%、3.1%。减少议事协调机构134个，精简41.8%。扎实开展扶贫领域腐败专项督导和惠民财政补贴资金“一卡通”专项审计，教育、民政、国土、卫生领域开展“4+X”系统治理取得成效。围绕经济发展重点领域开展跟踪审计，全面实施预算绩效管理，全年实现节支2.2亿元。

法治意识进一步增强。自觉接受人大监督，建立市政府重大决策事前向市人大常委会报告制度，办理人大代表建议150件，满意和基本满意率100%。自觉接受政协监督，定期向市政协常委会通报工作，办理政协提案208件，满意率100%。配合市人大有序推进立法工作。坚持政府常务会会前学法，重大决策专家咨询制度化，向社会公布市本级行政权力清单，全省首创政府“四本预算”全面公开。出台行政机关负责人出庭应诉规定。

各位代表，上下同欲者胜，民心凝聚者强。过去一年的成绩来之不易，这得益于以习近平同志为核心的党中央的英明领导，得益于习近平新时代中国特色社会主义思想的正确指引，得益于省委、省政府和市委的坚强领导，得益于全市上下的共同努力。在此，我谨代表市人民政府，向人大代表、政协委员、各民主党派、工商联、无党派人士、各人民团体，向人民解放军指战员、武警官兵、政法干警和民兵预备役人员，向全市人民，向坚决支持眉山政府工作的广大网友，表示崇高的敬意和衷心的感谢！

各位代表，前进的道路从来不会一帆风顺。我们也清醒认识到，眉山经济社会发展不平衡不充分的问题依然突出：经济发展质效不高不优的现象仍较明显；生态环境保护治理任重道远；民生领域还有不少短板，教育、医疗、养老等欠账较多，公共服务有待加强；社会治理还有薄弱环节，社会文明水平还需提高；开放发展意识还需深化，苦干兴眉精神还需加强，政府职能转变还需进一步到位。对此，我们将直面问题，敢于担当，尽心竭力加以解决。

二、2019年工作安排建议

2019年是中华人民共和国成立70周年，是深入学习贯彻习近平新时代中国特色社会主义思想和党的十九大精神的重要一年，是决胜全面建成小康社会的关键之年，是全面贯彻省委十一届三次、四次全会和市委四届十一、十二次全会部署的落实之年，也是市委确定的开放发展突破年。做好今年工作，意义重大，任务艰巨。

今年全市经济社会发展的主要预期目标是：全市地区生产总值增长7.8%左右，全社会固定资产投资增长10%以上，社会消费品零售总额增长10%以上，地方一般公共预算收入同口径增长7.5%左右，城乡居民人均可支配收入分别增长8%、9%，城镇登记失业率4.3%以内，居民消费价格指数涨幅控制在3%左右。全面完成省定安全生产和节能减排目标。

为此，我们将重点抓好五个方面工作：

（一）坚持开放引领，加快形成眉山开放发展新示范。

提升对外开放大平台。举全市之力加快眉山天府新区高质量发展，全方位提升眉山天府新区能级，聚力发展电子信息、数字经济、装备制造、先进材料、高端服务和总部经济，全力推进重大项目建设，确保片区规上工业增加值、固定资产投资分别增长9.5%、17.5%以上。高水平规划建设海峡两岸产业合作区眉山产业园。创建四川自贸区眉山协同改革先行区，争取海关眉山驻点设置，积极推进设立海关特殊监管区域及场所。加快眉山高新区创建国家级高新区、视高园区创建省级高新区。高水平协助联合国教科文组织筹办“历史村镇的未来”国际会议，支持青神县、东坡区办好竹博会、泡博会等重大会节活动。

加速成眉同城大突破。扎实推进《成眉同城化发展五年行动计划（2018—2022年）》。启动成眉同城化总规、成眉天府新区毗邻地区协同专规编制。推动成眉铁路公交化运营，加快红星路南延线等5条干线公路建设，加快眉山东环高速公路前期工作。启动成眉20平方公里高新技术转化基地建设。年内落地成都优质产业项目10个以上。引进一批成都优质医疗卫生、教育体育、文化艺术等资源进入眉山。推动成眉生态环境联防联控联治。

精准招引推进大项目。紧扣产业链、技术链、资本链关键环节，紧盯“三类500强”、行业龙头企业和具有独立知识产权的中小企业，打好各类招商“组合拳”，分层分类精准招商，力争年内引进“三类500强”或投资5亿元及以上项目30个，到位资金720亿元。强化要素保障，加快推进联想叠云创新科技园、川港合作示范园、中法农业科技园、恒大童世界、乐高乐园等150个省市重点项目建设，完成年度投资378亿元以上。

构建立体开放大通道。继续实施千亿交通大会战，全年完成投资150亿元以上。加快“三横七纵”高速路网和“两横八纵六联两环”干线路网布局，确保太和大道、岷东大道永寿至青神城区段、工业大道谢家至青龙段完工。完成新（改）建农村公路200公里，修好村民回家“最后一公里”。加快川藏铁路眉山段、市域铁路S5和S14线、洪雅二类通用机场前期工作。推进汤坝、虎渡溪航电枢纽工程建设。深入推进公交都市创建，建成城南综合客运枢纽站。加快打造蓉欧班列眉山现代物流港。

（二）坚持创新驱动，加快培育眉山高质量发展新动能。

突出稳企业，强力扶持民营经济。旗帜鲜明“大抓实体企业、大兴民营经济”，毫不动摇支持民营经济创新裂变，切实抓好“民营20条”“工业10条”等政策落地落实，深化企业全生命周期政策服务，让眉山企业家更有获得感、公平感、尊严感，民营经济增加值增长8%。加强企业梯队建设，围绕“3135”产业开展企业“建档立卡”“一企一策”精准扶持，支持眉山企业“小升规”“规改股”“股上市”，推动培育一批瞪羚企业，加快形成一批眉山本土大企业大集团，推动60户民营企业全面巩固现代企业制度。力争新增规上企业50户，10亿企业达到15户，新登记注册个体工商户1.2万户、私营企业2400户。做响做大眉山品牌，积极帮助企业拓展国内国际市场。建立领导干部联系重点产业、重点企业和行业协会商会制度，构建亲清新型政商关系。

突出增动能，强力加快“眉山智造”。实施新一轮大规模企业技术改造，推进设备换芯、生产换线、产品换代，力争工业投资、更新改造投资均增长10%以上。大力发展高新技术产业，培育国家高新技术企业5家，全市高新技术产业主营业务收入增长12%以上。加快发展数字经济，重点培育云计算、大数据等前沿产业，加快华为公有云西南节点等重点项目建设。加强科技成果转化转移，推进10项重点领域“硬核”科技成果转移转化示范项目。开展规上企业“专利消零行动”，力争重点培育企业研发活动覆盖率90%以上，研发投入增长10%。重点培育电池与材料、紧固件制造业创新中心。加快推进信利6代线、亚洲铝业西南基地、方大炭素、万华化学等智能制造项目，确保千禾味业年产25万吨酿造酱油和食醋生产线（一期）、凯盛光伏铜铟镓硒薄膜太阳能、川金象DMPP增效肥等一批重点项目建成投产，战略性新兴产业占规上工业总产值比重提高到25%。

突出添活力，强力推进重点改革。认真贯彻“巩固、增强、提升、畅通”八字方针，不断深化供给侧结构性改革，全面落实国家实施更大规模减费降税各项政策举措。以机构改革为新起点，纵深推进“放管服”改革，努力实现流程最优，加快推进“最多跑一次”改革和“一窗进出，一网通办”改革，网上可办率达到90%以上；努力实现机制最活，深化“多证合一”“多评合一”“多图联审、联合验收”模式；推广“红色代办”“模拟审批”，创新事中事后监管机制，力争营商环境主要评价指标达到国内一流水平。加快社会信用体系建设，健全守信联合激励和失信联合惩戒机制，扎实清理拖欠民营企业、中小企业账款等突出问题。增强金融服务实体经济能力，加快建立普惠金融体系，引导更多金融活水流向实体经济。加快国有平台公司整合，积极稳妥推进国有企业股权结构改革。深化财税体制改革，严格政府债务管理，坚决遏制隐性债务增量，积极妥善化解存量。

（三）坚持协调平衡，加快推动眉山城乡统筹新突破。

加快县域经济发展。深入实施“做强一带、做活一区、做美一片”战略，进一步完善推动县域经济高质量发展的支持政策和考核办法，强化分类指导和奖励政策，加快形成“三区协同、点极突破”发展格局。探索构建县域之间协同并举、利益共享的发展机制，推动东坡区、彭山区、岷东新区融合发展。深化扩权强县、扩权强镇改革，赋予基层更多自主权。

加快城市提升发展。巩固提升“四城联创”成果，建成国家森林城市，推动眉山城市品质更高、功能更强、颜值更靓。加速新型城镇化步伐，完成城镇基础设施投资200亿元，新增建成区4.2平方公里，常住人口城镇化率提高1.6个百分点。采取精准实用政策，加强房地产市场监管，促进房地产市场平稳健康发展。坚持城市留白理念，加强城市天际线、主色调和显山露水视线通廊的建设引导。统筹推进道路改造、地下管网等市政工程建设。积极推进“智慧城市”建设，提高城市精细化管理水平，大力整治“五乱”，科学规划中心城区交通，治理城市堵点。用政策引导推进既有住宅增设电梯。推动出台《眉山市物业管理条例》。推进公园城市建设，再新建公园12个。加强全民健身场地设施建设，规划建设乒乓球场、羽毛球场、足球场、篮球场等公共运动场地。实施全域安全饮水工程，完成市第三水厂一期工程并启动二期工程，建成向彭山区供水及“引青入东”“引青入丹”供水工程。启动第二自来水厂向青神县供水工程。

加快乡村振兴发展。以加快建设现代农业园区为牵引，大力深化农业供给侧结构性改革，建成1个省级现代农业产业园区。深入推进“味在眉山·香飘世界”行动，办好中国美食节（四川站）活动，做大做响竹编、泡菜、蜂蜜、晚熟柑橘等区域性主导产业品牌，力争泡菜产业销售收入达到200亿元，晚熟柑橘种植面积达到70万亩，“味在眉山”实现销售收入910亿元。决不放松粮食生产，耕地面积决不能减少，粮食产量稳定在120万吨以上。聚焦农村更美，建设美丽乡村竹林风景线，推进“美丽眉山·宜居乡村”建设，开展农村人居环境整治三年行动，深化农村垃圾、污水、厕所“三大革命”，扩大农村生活垃圾分类治理“百村示范”成果。健全自治、法治、德治相结合的乡村治理体系，传承发展优秀乡土文化，培育文明乡风、良好家风、淳朴民风。加快培育“五支队伍”，积极吸引各类人才流向农村。

加快文旅融合发展。全力推进黑龙滩国际生态旅游度假区、瓦屋山旅游整体开发等重点项目建设，完成瓦屋山国家4A级景区创建。打造“慢生活、亲山水、品乡愁”的乡村旅游业态。积极探索“旅游+产业”，注重与康养、体育、研学等多要素融合，打造一批具有眉山特色的旅游体验项目和文旅综合体，推进眉山全域旅游。进一步拓展旅游客源市场，加大三苏祠、黑龙滩、柳江古镇等旅游产品宣传推介，力争全市实现旅游收入450亿元左右。加快建设国家历史文化名城，不断满足市民日益增长的精神文化需求，提高眉山文化影响力和城市文化软实力。强力推进东坡文化传承创新“七个一”工程，全力打造三苏祠精品博物馆、苏东坡题材舞台剧，推进话剧《苏东坡》巡演，建设眉山东坡书院。办好2019年眉山东坡文化国际学术高峰论坛、四川音乐周、城市音乐季等重大文化活动。推进江口沉银博物馆和遗址公园建设。

（四）坚持绿色高质，加快书写眉山宜居美丽新画卷。

拓展绿色空间。深入推进“绿海明珠、千湖之城、百园之市”建设，扎实推进中央、省环保督察及“回头看”反馈问题整改。打好蓝天保卫战，确保PM2.5较基准年下降25%，空气优良天数比例达到75%。打好碧水保卫战，加快推进重点小流域治理，流域水质达到地表水Ⅳ类标准。加快环保基础设施建设，建成市第二污水处理厂一期工程并启动二期工程，205座污水处理设施全部建成。实施重点区域土壤整治和城市污染场地治理工程，加强工业固体废物处置监管，规范生活垃圾收集、转运和无害化处置。

坚持绿色发展。做大绿色经济，支持眉山天府新区打造绿色园区，积极培育指导企业创建省级以上绿色体系，新增省级绿色工厂3户以上。发展绿色餐饮，推进“家政无忧”工程，促进餐饮、家政、会展等行业提质升级。加快推进生态环境损害赔偿制度改革，严把投资眉山项目环保准入关，以透支环境为代价的项目再大也不引，影响眉山可持续发展的产业再多也不要。严格环保执法监管，开展自然资源资产审计，推进“大棚房”问题和重点区域违规违法占用国家资源专项清理整治。实行环境风险全过程管控，提升环境突发事件应急处置能力。倡导简约适度、绿色低碳生活方式，开展创建节约型机关、绿色学校、绿色社区、绿色出行等活动，新增公交、公务用车使用新能源车比例不低于30%、50%。

（五）坚持共建共享，加快实现眉山人民美好生活新向往。

努力让群众享有更高的收入。扎实做好创业就业工作，开展“三会一展”活动，全年就业培训不低于1.5万人次，新增就业不低于2.78万人。加强农民工服务保障，开展返乡农民工春节大走访活动。

努力让群众享有更满意的教育。深化教育改革，全面推进素质教育。实施学前教育新三年行动计划，2019年新（续）建齐通路幼儿园等公办幼儿园22所，竣工投用16所，学前三年毛入园率达到93%以上。加快推进西北片区中小学等城区学校建设10所，建成投用8所，新增学位1.4万个。加强高中基础设施建设和考点建设。全年新（改）建校舍15万平方米。大力引进和培育一批全国全省优质品牌学校，推动与香港大学、北京外国语大学、四川大学战略合作协议落地落实。支持在眉高校加快发展，加强应用型技能型人才培养。建设学习型城市，办好继续教育、特殊教育、家长学校。加强教师队伍建设，保障教师待遇，表扬一批优秀教师、校（园）长和“眉州名师”，让尊师重教在眉山蔚然成风！

努力让群众享有更高水平的健康服务。推进医药卫生体制改革，建设城市医疗集团和县域医联体，加快建立现代医院管理制度，促进分级诊疗。实施医卫腾飞行动计划，加快华西第二医院眉山妇女儿童医院等项目建设，确保市人民医院二期工程建成投用、仁寿县妇女儿童医院项目主体完工。推进公共卫生服务均等化，强化艾滋病、血吸虫病等重大疾病防控。培育本土医卫人才，引进外来医疗专家，加强全科医生培养，加快中医药产业发展，加强院长和院级管理团队建设。依法打击“医闹”，维护正常医疗秩序。加速医养健康深度融合，加快七里坪省级中医药健康旅游示范基地、彭祖国际健康养老养生文化城等项目建设。强化食品药品和保健品市场规范管理，决不允许打着保健品旗号坑害眉山老百姓。

努力让群众享有更好的社会保障。深入实施全民参保计划，严格落实被征地农民社会保障政策，加大失业保险基金支持企业稳定岗位力度。提升城乡住房保障水平，实行公共租赁住房实物配租和租赁补贴相结合的保障方式，稳步扩大住房公积金制度覆盖面。加强对特定人群特殊困难的精准救助，开展“救急难”综合试点。抓好未成年人保护工作，深化留守儿童关爱“童伴计划”。持续做好退役军人服务管理。深化养老服务体系建设，进一步提升养老服务质量，开展特殊老年人家庭设施适老化改造和老年人助餐服务点建设试点。加强公益性公墓建设，扎实推进绿色殡葬综合改革。

努力让群众享有更安定的社会环境。深入推进“平安眉山”建设，纵深推进扫黑除恶专项斗争，严厉打击电信诈骗、校园不良网络借贷等各类违法犯罪行为，提升社会治安满意度。持续抓好寄递物流、危爆物品、禁毒防艾等问题专项整治。抓好应急救援队伍建设，提高应急处置能力。严格落实安全生产责任制，坚决防范遏制重特大事故发生。推进“枫桥经验”眉山实践，推动多元解纷机制融合联动。完善公共法律服务体系建设。落实信访工作责任制，依法处置信访突出问题，化解一批信访积案难案。深化社会稳定风险评估工作，维护社会和谐稳定。

努力让脱贫群众稳定脱贫不返贫。锁定“两不愁、三保障”和“四个好”目标，实施“十大扶贫提升行动”和“两大对口帮扶行动”。坚持开展“回头看”“回头帮”，做到脱贫不脱帮扶、脱贫不脱政策、脱贫不脱项目。

努力让民生工程惠及更多群众。坚持民生投入和为老百姓办实事只增不减，全部财政性支出用于民生的比例不低于80%。紧紧围绕群众最关心、最直接、最现实的利益问题，全力推进人大代表票决民生实事项目和其他民生项目，让改革发展成果更多更公平惠及全市人民，不断满足群众日益增长的美好生活需求。

努力让各项社会事业全面发展。统筹抓好国防动员、双拥、人民防空、统计、民族、宗教、保密、档案、气象、测绘、供销、红十字会、慈善、妇女、残疾人、关心下一代等工作，推动各项事业发展进步。

三、大力加强政府自身建设

聚焦新目标、落实新部署，必须担当新使命，展现新作为。新的一年，我们将勤勉履职、锐意进取，严格落实全面从严治党要求，不断加强政府自身建设，着力提升政府执行力和公信力，努力建设人民满意的服务型政府。

（一）坚持从严治政，建设政治坚定、干净干事的清廉政府。始终坚持以习近平新时代中国特色社会主义思想为指引，树牢“四个意识”，坚定“四个自信”，坚决做到“两个维护”。加强党的领导，坚持重大事项向市委常委会报告制度。落实全面从严治党主体责任，推动政府系统党风廉政建设纵深发展。深化廉政风险防控，构建统一高效审计监督体系，做好巡视发现问题整改，管好用好公共资金、公共资源、国有资产，严防不正之风和腐败问题发生，大力营造风清气正干事环境。

（二）坚持为民勤政，建设开拓创新、有为高效的务实政府。越是困难的时候，我们越要在市委的坚强领导下，始终和企业、职工群众想在一起、站在一起、干在一起，做好充分准备，有所为有所不为，从容应对风险挑战。坚持“一线工作法”，推进市政府领导班子成员联系企业、联系项目、联系区县，推进各级政府和部门“一把手”深入基层、深入一线，切实解决发展需要、群众急盼的实际问题。把群众和企业满意作为政府服务的第一标准，全面开拓创新，运用大数据、物联网等技术推动政府管理创新，持续推进“互联网+政务服务”，按“最小颗粒度”推进办事指南标准化、场景化，着力推进跨层级、跨部门信息互认共享。坚持政府过紧日子，今年一般性行政支出减少5%，从严控制“三公”经费开支，腾出更多的财力用于民生事业。

（三）坚持依法行政，建设厉行法治、公开透明的法治政府。全面落实法治政府建设实施纲要，自觉运用法治思维和法治方式推动各项工作。健全行政决策机制，建立法律顾问参与规范性文件备案审查制度，对涉及重大公共利益事项，认真听取民主党派、工商联、无党派人士、人民团体等社会各界意见，认真倾听网民诉求，继续坚持“开门听音·网络问政”。认真执行市人大及其常委会的决议决定，自觉接受法律监督、工作监督，办好人大代表建议。主动接受政协民主监督，推进政府协商，办好政协提案。主动回应社会关切，接受监察监督、司法监督、群众监督和舆论监督。深入推进政府“五公开”，让权力在阳光下运行。坚持诚信施政，力戒形式主义、官僚主义，定下的事情说干就干，认准的事情一抓到底！

各位代表，新时代孕育复兴希望，新征程激发奋斗力量。让我们更加紧密地团结在以习近平同志为核心的党中央周围，在省委、省政府和市委的坚强领导下，主动担当、主动服务，锐意进取、科学作为，全心全意为群众谋幸福，尽力尽责为眉山谋发展，奋力谱写眉山发展壮丽新篇章，以优异成绩迎接中华人民共和国成立70周年！
